# Water immersion of dogs close to the time of topical fluralaner treatment does not reduce efficacy against a subsequent experimental challenge with *Rhipicephalus sanguineus* (*sensu lato*)

**DOI:** 10.1186/s13071-017-2389-9

**Published:** 2017-09-25

**Authors:** Heide Dongus, Leon Meyer, Rob Armstrong

**Affiliations:** 1 MSD Animal Health, Intervet Deutschland GmbH, Feldstraße 1a, 85716 Unterschleißheim, Germany; 2 Clinvet International, Uitzich Road, Bainsvlei, Bloemfontein, 9338 South Africa; 3 Merck Animal Health, 2 Giralda Farms, Madison, NJ USA

**Keywords:** Bathing, Bravecto, Dog, Fluralaner, *Rhipicephalus sanguineus*, Water

## Abstract

**Background:**

Fluralaner is a novel systemic ectoparasiticide for dogs and cats providing immediate and persistent flea- and tick-control after a single topical dose. Prescribing directions recommend waiting 72 h following topical administration before immersing dogs in water. The objective of this study was to determine whether water immersion immediately prior to treatment or earlier than 72 h post-treatment reduced subsequent treatment efficacy.

**Methods:**

Forty (*n* = 40) dogs were blocked on tick carrying capacity into 5 experimental groups and all but one of the groups (untreated control) were treated topically with fluralaner (Bravecto® Spot-On Solution, Merck Animal Health, Madison, NJ, USA) at the commercial dose. Three of the four remaining groups were immersed in 38–40 °C water for a 5 min bath - either 1 h before treatment; 12 h after treatment; or 24 h after treatment. Seven days after treatment all dogs were challenged with 50 *Rhipicephalus sanguineus* (*sensu lato*) ticks and after 24 h attached ticks were counted and removed.

**Results:**

Efficacies (compared to the untreated control group) were: 99.3% for no water immersion; 99.6% for immersion 1 h before treatment; 99.3% for immersion 12 h after treatment; and, 100% for immersion 24 h after treatment.

**Conclusions:**

Water immersion of dogs around the time of topical fluralaner administration did not reduce subsequent systemic acaricidal efficacy.

## Background

Fluralaner is a novel isoxazoline class compound that provides fast and persistent insecticidal and acaricidal efficacy in several formulations for dogs or cats [1]. Topically administered fluralaner is absorbed transdermally following administration [2], and a single dose provides 12 weeks of systemic control against multiple flea and tick species [1]. This insecticidal and acaricidal efficacy is not reduced by water and shampoo exposure after 72 h following treatment [3].

Veterinarians and/or dog owners may inadvertently immerse a dog in water within 72 h of treatment, or a dog may make its own choice to get wet, in spite of the precaution in the prescribing information [1]. Another possibility is that dogs bathed shortly before treatment may still have wet skin and hair at the time of treatment. The objective of this study was to determine whether either of these situations reduces subsequent acaricidal efficacy of topically administered fluralaner.

## Methods

Forty (*n* = 40) healthy mixed-breed dogs that had not received prior insecticide or acaricide treatment for at least 12 weeks were ranked on *Rhipicephalus sanguineus* (*sensu lato*) carrying capacity in a pre-treatment tick challenge and then randomly blocked into five groups (Table 1) without regard to gender. Four groups of dogs were treated with topical fluralaner (Bravecto Spot-On Solution, MSD Animal Health, Madison, NJ, USA) at the recommended label dose (25–56 mg/kg) based on the dog’s weight. In this study, dogs weighing between 10 and 20 kg received 500 mg fluralaner and dogs weighing between 20 and 40 kg received 1000 mg fluralaner. Three of these groups were also immersed for 5 min in fresh lukewarm (38–40 °C) water (using new water for each immersion time point) 1 h before, 12 h after, or 24 h after topical fluralaner treatment (Table 1). Seven days after topical fluralaner administration, all dogs in all groups were challenged with 25 adult male and 25 adult female unfed *R. sanguineus* (*s.l*.) ticks. The dogs were sedated for approximately 1 h and placed in an infestation chamber to reduce stress to the dog and to facilitate tick infestations by allowing ticks to attach. After 24 h, forceps were used to remove and count all ticks from all dogs.

**Table 1:** Treatments of five groups of dogs to evaluate the impact on efficacy of water immersion close to the time of topical fluralaner treatment

Group (*n* = 8)	Treatment
Untreated control	No treatment and no water immersion
Positive control	Treatment with topical fluralaner and no water immersion
Pre-treatment immersion	Water immersion 1 h before treatment with topical fluralaner
Post-treatment immersion (12 h)	Treatment with topical fluralaner followed by water immersion after 12 h
Post treatment immersion (24 h)	Treatment with topical fluralaner followed by water immersion after 24 h

Efficacy (%) was calculated by comparing mean tick numbers (arithmetic means were used for all calculations) on each treated group with mean tick numbers on the untreated control group using the formula 100 × (MC – MT) / MC, where MC is the mean number of live attached ticks on untreated control dogs and MT is the mean number of total live attached ticks on dogs in each fluralaner treated group. The experimental unit was the individual dog and the groups were compared using an ANOVA (SAS Proc GLM procedure) with a treatment effect on both untransformed and logarithmic transformed tick (count +1) data.

## Results

The mean acaricidal efficacy of topical fluralaner in all of the treated groups was between 99.3% and 100% (Table 2). There were no significant differences between the number of ticks on any fluralaner treated group (all *P*-values > 0.22) and every fluralaner-treated group had significantly fewer ticks than the untreated control group (Table 2). No adverse event was observed in any treated dog.

**Table 2:** Acaricidal efficacy (24 h) against *Rhipicephalus sanguineus* (*sensu lato*) on dogs 7 days after topical fluralaner treatment and water immersion at various times

Treatment	Acaricidal efficacy (%)	*P*-value vs untreated control	F_(4,35)_ value vs untreated control	Live attached ticks (mean ± SD)
Topical fluralaner	99.3	< 0.0001	98.02	0.3 ± 0.5
No treatment	na	na	na	34.5 ± 9.8
Water immersion 1 h *before* topical fluralaner treatment	99.6	< 0.0001	98.02	0.1 ± 0.4
Water immersion 12 h *after* topical fluralaner treatment	99.3	< 0.0001	98.02	0.3 ± 0.7
Water immersion 24 h *after* topical fluralaner treatment	100	< 0.0001	98.02	0.0 ± 0.0

*Abbreviations: na* not available, *SD* standard deviation

## Discussion

Water immersion had no impact on subsequent acaricidal efficacy, either from immersion 1 h prior to administration or from immersion 12 to 24 h after topical fluralaner administration. This outcome may not be surprising, given that topically administered fluralaner is absorbed across the skin and subsequently delivers its acaricidal effect through systemic distribution which would not be affected by water immersion. However, water immersion could reduce subsequent efficacy of a systemic active ingredient, for example: immersion before treatment could reduce transdermal absorption by increasing product run off or drip off at the application site. Water immersion post-treatment could wash active ingredient away from the application site with consequent reduction in plasma levels through decreased transdermal active ingredient absorption. These results show there was no efficacy reduction following water immersion at the time point tested and is an indication that there was little or no loss of active ingredient from the administration site.

Tick challenges in this study were administered 7 days after treatment and lasted for 24 h. The pharmacokinetic (PK) pattern of absorbed fluralaner after topical administration to dogs at the minimum clinical dose shows that 7 days is just prior to the expected C_max_ plateau; therefore, a 24 h *R. sanguineus* (*s.l*.) challenge at this time provides relevant data showing that sufficient fluralaner was absorbed to provide effective systemic acaricidal protection [2]. A 24 h tick challenge time was used rather than a 48 h challenge time as in the previous water immersion study [3] to increase the severity of the challenge. Dogs in this study were not treated with shampoo as in the previous study [3], and intentional shampoo use within 72 h after topical fluralaner administration is not recommended.

Water immersion 1 h before treatment is consistent with the product prescribing information [1]. The high acaricidal efficacy observed in dogs immersed before treatment indicates that a dog that swims shortly before topical fluralaner administration is not likely to experience a subsequent reduction in acaricidal efficacy.

## Conclusions

Water immersion of dogs 1 h before or more than 12 h after topical fluralaner treatment did not reduce subsequent systemic acaricidal efficacy.
